# Is avolition in schizophrenia associated with a deficit of dorsal caudate activity? A functional magnetic resonance imaging study during reward anticipation and feedback

**DOI:** 10.1017/S0033291714002943

**Published:** 2015-01-12

**Authors:** A. Mucci, D. Dima, A. Soricelli, U. Volpe, P. Bucci, S. Frangou, A. Prinster, M. Salvatore, S. Galderisi, M. Maj

**Affiliations:** 1 Department of Psychiatry, University of Naples SUN, Naples, Italy; 2 Psychosis Research Program, Department of Psychiatry, Icahn School of Medicine at Mount SinaiNew York, USA; 3 MRC Social Genetic and Developmental Psychiatry, Institute of Psychiatry, King's CollegeLondon, UK; 4 University of Naples ‘Parthenope’ and IRCCS Research Institute SDN, Naples, Italy; 5 Biostructure and Bioimaging Institute, National Research Council, Naples, Italy; 6 Department of Biomorphological and Functional Studies, University of Naples ‘Federico II’, Naples, Italy

**Keywords:** Avolition, deficit schizophrenia, dorsal caudate, reward anticipation, schizophrenia, ventral striatum

## Abstract

**Background:**

The neurobiological underpinnings of avolition in schizophrenia remain unclear. Most brain imaging research has focused on reward prediction deficit and on ventral striatum dysfunction, but findings are not consistent. In the light of accumulating evidence that both ventral striatum and dorsal caudate play a key role in motivation, we investigated ventral striatum and dorsal caudate activation during processing of reward or loss in patients with schizophrenia.

**Method:**

We used functional magnetic resonance imaging to study brain activation during a Monetary Incentive Delay task in patients with schizophrenia, treated with second-generation antipsychotics only, and in healthy controls (HC). We also assessed the relationships of ventral striatum and dorsal caudate activation with measures of hedonic experience and motivation.

**Results:**

The whole patient group had lower motivation but comparable hedonic experience and striatal activation than HC. Patients with high avolition scores showed lower dorsal caudate activation than both HC and patients with low avolition scores. A lower dorsal caudate activation was also observed in patients with deficit schizophrenia compared to HC and patients with non-deficit schizophrenia. Dorsal caudate activity during reward anticipation was significantly associated with avolition, but not with anhedonia in the patient group.

**Conclusions:**

These findings suggest that avolition in schizophrenia is linked to dorsal caudate hypoactivation.

## Introduction

Avolition, i.e. a deficit of motivation, is highly prevalent in schizophrenia, being regarded as a key aspect of the negative syndrome (Kirkpatrick *et al.* 2006; Foussias *et al.* 2009; Strauss *et al.* 2013*a*). It can be found already in the prodromal stage of the disorder, and is often reported to be an important predictor of poor outcome (Foussias *et al.* 2009; Strauss *et al.* 2013*b*).

Advances in cognitive and affective neuroscience have informed the current conceptualization of motivation as a multifaceted construct, including hedonic experience (i.e. the ability to enjoy in-the-moment pleasant experience, also referred to as ‘liking’), reward prediction (i.e. the ability to motivate behavior to achieve an expected, but not currently available pleasant experience), and other distinct elements, such as reward valuation, effort valuation, encoding of action-outcome contingency, and decision-making processes.

The activity of partially independent cortico-striatal circuits seems to subtend different aspects of motivation, which should be regarded as separate, although interrelated, components (Wallis, 2007; Barch & Dowd, 2010; Der-Avakian & Markou, 2012; Miller *et al.* 2014). In fact, studies in healthy individuals have shown that a brain network including the ventral striatum, orbito-frontal cortex (OFC), insula, and medial prefrontal cortex (mPFC) is involved in some aspects of motivation, such as liking, reward anticipation, reward valuation, and representation of stimulus-reward associations, while a circuit including the dorsal caudate and dorsolateral prefrontal cortex (DLPFC) underlies other aspects of motivation, such as encoding of action-outcome contingency and representation of the expected reward value of action (Berridge & Robinson, 2003; Delgado *et al.* 2005; Haruno & Kawato, 2006; Balleine *et al.* 2007; Wallis, 2007; Barch & Dowd, 2010; Haber & Knutson, 2010). These different facets of motivation have synergic functions in instrumental learning and adaptive behavior (Dolan & Dayan, 2013).

Current research suggests that persons with schizophrenia have intact in-the-moment hedonic experience (liking), but show abnormalities in other facets of the motivational system (Gard *et al.* 2007; Heerey & Gold, 2007; Heerey *et al.* 2007; Waltz *et al.* 2007; Kring & Moran, 2008; Barch & Dowd, 2010; Cohen & Minor, 2010; Foussias & Remington, 2010; Simpson *et al.* 2012; Mann *et al.* 2013; Strauss *et al.* 2013*a*). Most brain imaging research has focused on reward prediction deficit, reporting that a ventral striatum dysfunction is the neurobiological substrate of that deficit in patients with schizophrenia treated with first-generation antipsychotics (FGAs) or unmedicated/never medicated (Juckel *et al.* 2006*a*,*b*; Schlagenhauf *et al.* 2009; Nielsen *et al.* 2012*a*). However, no deficit of ventral striatum activity during reward prediction was found in patients treated with second-generation antipsychotics (SGAs), despite the presence of avolition in the same patients (Juckel *et al.* 2006*b*; Schlagenhauf *et al.* 2008; Walter *et al.* 2009; Nielsen *et al.* 2012*b*). Furthermore, ventral striatum hypoactivation has been found to correlate not only with measures of avolition (Simon *et al.* 2010) or avolition plus anhedonia (Waltz *et al.* 2010), but also with measures of depression (Simon *et al.* 2010) or positive symptoms (Nielsen *et al.* 2012*a*, *b*; Esslinger *et al.* 2012).

No study has focused as yet on the circuit involving the dorsal caudate and the DLPFC during reward anticipation in persons with schizophrenia, in spite of accumulating evidence that this circuit plays a key role in motivation (Palmiter, 2008; Balleine & O'Doherty, 2010; Wang *et al.* 2013; Miller *et al.* 2014).

In the present functional magnetic resonance imaging (fMRI) study, using the Monetary Incentive Delay (MID) task (Knutson *et al.* 2000), we investigated: (*a*) the activation of the ventral striatum and dorsal caudate during anticipation of reward or loss in patients with schizophrenia living in the community and stabilized on treatment with SGAs only, (*b*) the relationships of ventral striatum and dorsal caudate activation with hedonic experience and motivation, and (*c*) differences in striatal activation of patients with high and low avolition scores, as well as of patients with Deficit Schizophrenia (DS), characterized by primary and persistent negative symptoms, and Non-Deficit schizophrenia (NDS) which might have different reward sensitivity.

The study included chronic patients with schizophrenia, as this population might have a full range of persistent avolition severity, while showing attenuated or remitted positive symptoms.

## Method

### Subjects

All outpatients with a DSM-IV diagnosis of schizophrenia attending the outpatient unit of the Department of Psychiatry of the University of Naples SUN between September 2010 and July 2012 were screened for the study. Diagnoses were confirmed using the Mini International Neuropsychiatric Interview-Plus (MINI-Plus), a structured interview for DSM-IV and ICD-10 diagnosis used in research settings (Sheehan *et al.* 1998). Additional eligibility criteria were: age between 18 and 65 years; no evidence of mental retardation; clinically stable (i.e. no hospitalization or change in psychotropic medication for 3 months prior to scanning), to avoid the presence of severe positive symptoms which might cause secondary negative symptoms; treatment with SGAs only; no history of head trauma with loss of consciousness; and no substance abuse or dependence in the preceding 6 months (except for smoking).

Sex- and age-matched (±3 years) healthy controls (HC) were recruited from the community via flyers and screened to exclude any lifetime personal history of mental illness using the MINI-Plus. Additional eligibility criteria were: no family history of mental illness or psychiatric hospitalization; no past history of head trauma with loss of consciousness; no lifetime history of substance abuse or dependence (except for smoking); not on prescribed medications that might affect CNS functions.

The study was approved by the University Ethics Committee, and all participants signed a written informed consent.

### Assessments

All subjects were interviewed to record socio-demographic variables, including age, education and socioeconomic status. An estimate of full-scale IQ was obtained using the revised version of the Wechsler Adult Intelligence Scale.

All participants completed the Temporal Experience of Pleasure Scale (TEPS; Gard *et al.* 2006), an 18-item self-report measure of anticipatory and consummatory pleasure, with higher scores indicating greater experience of pleasure; and the Revised Physical Anhedonia Scale (PAS; Chapman & Chapman, 1978), a 61-item scale evaluating trait anhedonia, with higher scores indicating greater anhedonia. Participants were also administered the Quality of Life Scale (QLS; Heinrichs *et al.* 1984). According to Nakagami *et al.* (2010), real-life motivation was computed as the average score on three QLS items: Motivation (‘ability to sustain goal-directed activities’), Curiosity (‘degree to which one is interested in his/her surroundings’), and Sense of Purpose (‘realistic integrated life goals’), with higher scores indicating greater motivation.

Patients were administered the Schedule for the Deficit Syndrome (SDS; Kirkpatrick *et al.* 1989), through which avolition was assessed by summing the scores on the items Curbing of Interests, Diminished Sense of Purpose, and Diminished Social Drive, with higher scores indicating greater avolition (Kirkpatrick *et al.* 1989; Kimhy *et al.* 2006; Galderisi *et al.* 2013); and the Positive and Negative Syndrome Scale (PANSS; Kay *et al.* 1987) to assess positive symptoms and depression. The daily antipsychotic dose on the day of scanning was converted to chlorpromazine equivalents following Gardner *et al.* (2010).

### Experimental design

We used a modified version of the MID task (Knutson *et al.* 2000) including 96 trials, each lasting 8 s, with a total task duration of 12 min. In this task, the subject has to press a button within a predefined time window to win or avoid losing money. There were four incentive conditions (18 trials each) – large reward, small reward, large loss, and small loss – and a neutral condition (24 trials), presented in a random order and indicated by a different cue (online Supplementary Fig. S1). The number of trials was chosen according to the original MID task studies (Knutson *et al.* 2000, 2001*a*, *b*) and several other studies carried out in patients with schizophrenia (Juckel *et al.* 2006*a*, *b*; Schlagenhauf *et al.* 2008). All these studies were able to demonstrate significant incentive effects in relatively small groups (about 10 subjects per group).

During each trial, participants were presented with one of the cues for 250 ms, followed by a fixation cross for 2000–2500 ms, and then by a white target for 160–360 ms. Subjects either gained or avoided losing money by pressing a button during the short target presentation time window. A feedback followed for 1650 ms, with the amount of money gained or lost in the trial and the cumulative outcome. The inter-trial interval was jittered between 3240 and 3940 ms to keep constant the trial duration (8 s).

The task difficulty was personalized during a practice session prior to scanning according to the original MID task design (Knutson *et al.* 2001*a*, *b*) to allow at least 66% of success. Task individualization was aimed to make the task difficulty comparable across subjects but not to oversimplify the task for patients. Patients were slower and target exposure was longer but the difficulty was set at the same level for patients and controls. In fact, target offset was individually determined based on the reaction time recorded during the practice session, so that each subject experienced difficulties in hitting the button in time before target offset in about 33% of the trials. The post-training target offset varied from 140 to 530 ms (92.9% of patients and 100% of HC were in a range from 166 to 450).

Subjects viewed the stimuli, projected onto a back-illuminated translucent screen, through a mirror attached to the head coil. They were instructed to press the button as fast as possible irrespective of the cue type. After the scan, participants were paid the amount of money they won.

Smokers were allowed to smoke prior to MRI scanning (last cigarette approximately 60 min before session) to avoid the potential effects of nicotine withdrawal.

### MRI acquisition parameters

Structural and functional images were acquired on a 3.0-T scanner (Philips, Achieva, The Netherlands), equipped with a standard radio-frequency head coil. Head movements were restricted using foam cushions. Structural images were acquired via a high resolution, T1-weighted 3D MPRAGE sequence (TR = 7.1 ms; TE = 3.2 ms; flip angle = 9°; voxel = 1 × 1 × 1 mm). T2*-weighted functional images covering the whole brain were acquired using a GRE-EPI sequence depicting an event-related blood oxygen-level dependent (BOLD ) signal (TR = 2000 ms; TE = 40 ms; thickness = 4 mm; matrix size = 128 × 128; FOV = 230 mm; voxel = 3.59 mm^2^), providing 32 interleaved images per volume, parallel to the AC–PC line and covering the whole brain. Each fMRI series consisted of 368 images, the first four of which were discarded to allow the scanner to reach a steady state.

### Image processing

For image preprocessing and GLM analysis, the SPM8 software package (Wellcome Trust Centre for Neuroimaging, London, UK; http://www.fil.ion.ucl.ac.uk) was used. Preprocessed images were corrected for differences in slice-time acquisition, realigned to the mean volume, and spatially normalized to the standard template of the Montreal Neurological Institute (MNI). The spatially normalized data were smoothed with an isotropic Gaussian filter (6 mm full-width half-maximum) to compensate for normal variation across subjects.

For each subject, data were modeled with a general linear model, with six movement parameters as nuisance regressors. Vectors of onset representing large reward, small reward, large loss, small loss, and neutral condition were convolved with a canonical hemodynamic response function. Contrast images of BOLD activity associated with incentive compared to neutral trials were produced for each participant.

Whole brain random-effects statistical maps were thresholded at *p* < 0.001, with an extent threshold of 10 voxels, uncorrected for multiple comparisons. False discovery rate (FDR) corrections for multiple comparisons were performed on all results, except for the ventral striatum and dorsal caudate, for which small volume statistics were applied, for which a family-wise error (FWE) peak correction was deemed appropriate, as these are considered appropriate for small structures, where a relatively small number of voxels per cluster is to be expected (Walter *et al.* 2009). Predefined volumes of interest (VOIs) for these structures were derived from key relevant publications. The MNI coordinates for the ventral striatum (x = ±9, y = 5, z = −2) were defined according to a recent study by Nielsen *et al.* (2012*b*). For the dorsal caudate (x = ±15, y = 8, z = 22), the coordinates were set according to Robinson *et al.* (2012), based on connectivity patterns. In each participant, VOIs were defined as cubes measuring 10 × 10 × 10 mm centered on the above coordinates. The shape and dimension of the region of interest were adopted from Nielsen *et al.* (2012*a, b*) who investigated ventral striatal activation in patients with schizophrenia and its associations with psychopathology. Online Supplementary Fig. S2 illustrates the VOI boundaries.

For correlations, measures of brain activation (mean parameter estimates within a VOI with equal weights for all voxels) for each contrast were extracted from the above VOIs as well as from VOIs of key cortical regions connected with ventral striatum and dorsal caudate, namely OFC and DLPFC, using the MARSBAR toolbox (http://marsbar.sourceforge.net).

### Statistical analyses

Group differences in sex distribution were assessed by the Pearson's χ^2^ test. Analysis of variance (ANOVA) was used to test group differences on continuous variables.

Relationships of measures of brain activation in the ventral striatum, dorsal caudate, OFC and DLPFC with anhedonia, motivation and avolition were examined in patients using Pearson's correlation coefficients. Intercorrelations between anhedonia and avolition/motivation measures were examined to verify whether these were distinct constructs or measured partially overlapping constructs. Differences in the correlation pattern between striatal VOIs (dorsal caudate and ventral striatum) or connected VOIs (DLPFC and OFC, respectively) were examined using Steiger's *Z* test, if at least one correlation with the scores for motivation/avolition or anhedonia was significant. Since multiple correlations were computed between VOIs and the above scores, a Bonferroni correction for multiple test was applied and will be reported; however, since Bonferroni correction can bias results toward type-2 statistical error, particularly for a relatively small sample size, and given our *a priori* hypothesis of the direction of the correlations (negative for avolition or anhedonia, and positive for motivation), only Bonferroni correction for one-tailed alpha = 0.05 will be reported (Bonferroni-corrected *p* = 0.008). Since antipsychotic treatment, depression severity and positive symptoms might influence striatal activity, anhedonia and motivation, correlations between these variables were examined using partial correlation analyses controlling for daily antipsychotic dose, positive symptoms scores and depression scores.

## Results

### Subject characteristics

Twenty-eight patients with schizophrenia and 22 HC completed the study. There was no group difference in sex, age, parental education, socioeconomic status or proportion of habitual cigarette smokers (Table 1). No current Axis I disorder other than schizophrenia was present in patients; a lifetime diagnosis of major depression, single episode, was present in only one patient, while a lifetime anxiety disorder was present in two patients (in one case panic disorder without agoraphobia and in the other obsessive compulsive disorder). Patients had significantly lower IQ and education than controls (Table 1). Since these variables can affect both performance on the MID task and subjects’ ability to report their experience on self-administered scales, education and IQ were entered as covariates in group comparisons on these measures and fMRI contrasts. There was no group difference in trait anhedonia as evaluated by the PAS, or anticipatory and consummatory experience of pleasure as assessed by the TEPS. Patients had significantly lower real-life motivation than controls. In the latter group, 18 out of 22 subjects had the maximum score of 6 and the remaining individuals had a score of 5, with a clear ceiling effect; among patients, two out of 28 reached the score of 6 and only five had a score of 5.

**Table 1.** Study sample: descriptive information

	HCs (*N* = 22)	SCZ (*N* = 28)	*F/χ*^2^	df	*p*
Demographic information					
Sex (M/F)	10/12	18/10	1.77	1	0.18
Age, years	31.91 ± 8.49	33.10 ± 6.67	0.29	1.48	0.59
Education, years	16.04 ± 2.69	12.43 ± 3.33	17.4	1.48	0.00001
Paternal education	11.16 ± 4.99	10.20 ± 5.09	0.16	1.48	0.69
Maternal education	10.00 ± 5.19	9.93 ± 4.94			
Socioeconomic status	35.71 ± 11.12	33.24 ± 14.03	0.44	1.46	0.51
General cognitive abilities					
WAIS-R Full-scale IQ	113.76 ± 15.23	79.35 ± 18.23	53.8	1.48	0.000001
Real-life motivation and anhedonia					
Real-life motivation	5.79 ± 0.36	3.16 ± 1.65	9.54	1.45	0.005
TEPS consummatory pleasure	4.84 ± 0.61	4.15 ± 0.81	0.23	1.43	0.63
TEPS anticipatory pleasure	4.58 ± 0.53	3.95 ± 0.63			
PAS total score	17.14 ± 6.32	23.80 ± 8.07	0.46	1.43	0.50
Clinical information					
PANSS Positive		8.04 ± 4.13			
PANSS Negative		11.64 ± 6.87			
PANSS Disorganization		7.11 ± 2.56			
PANSS Depression		2.53 ± 0.67			
SDS Avolition		4.64 ± 3.29			

HCs, Healthy controls; SCZ, subjects with schizophrenia; WAIS-R, Wechsler Adult Intelligence Scale – Revised; IQ, Intelligence Quotient; TEPS, Temporal Experience of Pleasure Scale; PAS, Revised Physical Anhedonia Scale; PANSS, Positive and Negative Syndrome Scale; SDS, Schedule for the Deficit Syndrome; Real-life motivation: average score on the Motivation, Curiosity and Sense of Purpose items of the Quality of Life Scale

### MID task performance

Details of MID task performance are shown in online Supplementary Table S1. For reaction time, a main effect of the cue was found in controls only (*F*_4,84_ = 8.12, *p* < 0.00001), due to the fact that they were faster for small reward (*p* < 0.002) and large reward (*p* < 0.00001) than for neutral cues. Patients did not show a significant effect of the cue (*F*_4,108_ = 0.84, *p* = 0.49). There was no main effect of diagnosis (*F*_1,46_ = 1.20, *p* = 0.28) and no cue × diagnosis interaction (*F*_4,184_ = 1.05, *p* = 0.39).

As expected, due to the personalized duration of the target presentation, there was no group difference in the number of successful trials and there was no significant cue × diagnosis interaction on the same measure. Accordingly, there was no group difference in the average monetary gain during the task.

Since there was no significant difference in behavioral data between large and small incentive cues, large and small reward trials were collapsed into a single reward condition, and large and small loss trials were collapsed into a single loss condition in all fMRI comparisons.

### fMRI results

#### Anticipation of reward or loss *v*. neutral

**Fig. 1.**
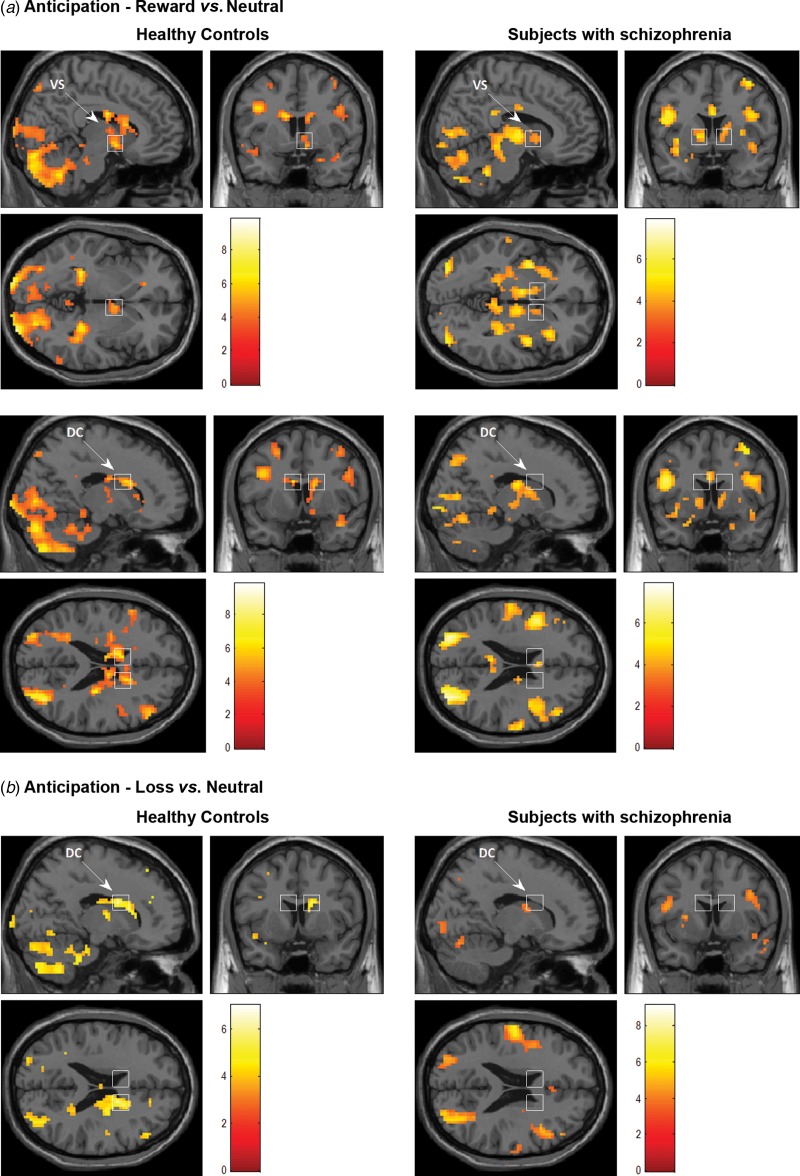
Mean blood oxygen level-dependent activity for the contrasts (*a*) reward *v*. neutral anticipation and (*b*) loss *v*. neutral anticipation in ventral striatum (VS, upper row, Talairach coordinates x = 9, y = 5, z = −2) and dorsal caudate (DC, middle and bottom rows, Talairach coordinates x = 15, y = 9, z = 20) in healthy controls (right sections) or subjects with schizophrenia (left sections). For illustrative purposes, maps were thresholded at *p* < 0.001, with an extent threshold of 10 voxels. Left is on the left (neurological convention).

For the contrast anticipation of the reward *v*. neutral condition, both patients and controls showed an activation in the ventral striatum, which was significant only on the right side in controls and on both sides in patients (Table 2 and Fig. 1). The dorsal caudate was significantly activated bilaterally in controls, but showed no activation in patients (Table 2 and Fig. 1). The key cortical regions involved in reward processing were also active in both groups, namely the OFC (BA 47, Table 2) and the DLPFC (BA 9/46, Table 2). As reported in Table 2, controls showed an activation of the right hippocampus and parahippocampal gyrus (BA 36), visual areas and cerebellum, while patients presented an activation in a larger number of cortical areas, including visual and cerebellar areas, right insula and temporal pole (BA 13/38) adjacent to the OFC, right anterior cingulate (BA 24/23), left inferior frontal (BA 44/45), as well as parietal areas (BA 43 and BA 7). However, no significant between-group difference for any of these regions was observed for that contrast.

**Table 2.** Functional magnetic resonance imaging activations during anticipation of reward *v*. neutral

		Healthy controls				Subjects with schizophrenia			
		Peak *t* test value	Talairach coordinates			Peak *t* test value	Talairach coordinates		
	BA		x	y	z		x	y	z
Right ventral striatum		5.38^a^	9	2	−4	4.01^a^	12	8	1
Left ventral striatum						4.91^a^	−12	2	1
Right dorsal caudate		6.00^a^	12	12	17				
Left dorsal caudate		6.35^a^	−12	6	20				
Right orbitofrontal cortex	47	3.92	53	19	−8	6.61	33	19	−8
Right temporal pole	38	5.20	45	19	−15	6.33	48	16	−15
Right dorsolateral prefrontal cortex	46	5.27	50	43	7	5.87	45	37	7
Right dorsolateral prefrontal cortex	9	4.57	48	9	25	5.64	53	21	25
Right insula	13					5.83	42	−1	−4
Right anterior cingulate gyrus	24					6.11	3	0	26
Right anterior cingulate gyrus	23					5.73	0	−29	30
Right hippocampus		8.21	27	−36	3				
Right parahippocampal gyrus	36	5.48	39	−31	−10				
Left inferior frontal gyrus	44					6.38	−45	12	20
Left inferior frontal gyrus	45					4.88	−42	20	14
Left precuneus/cuneus	19/18	9.88	−24	−100	−2	7.75	−30	−69	35
Right cerebellum declive		9.32	27	−80	−13	7.49	27	−66	−21
Right cerebellum culmen						6.25	3	−40	−22
Left inferior parietal lobule	40					6.79	−48	−42	45
Right superior parietal lobule	7					7.72	27	−66	46

BA, Brodmann area.

Brain activation during anticipation of reward. All results are significant at *p* < 0.05, false discovery rate corrected for multiple comparisons except when otherwise specified.

a Significant family-wise error corrected for small volume.

For the contrast anticipation of the loss *v*. neutral condition, the ventral striatum was not active in either group. The dorsal caudate was significantly activated bilaterally in controls, but showed no activation in patients (Table 3, Fig. 1). The DLPFC was activated in both groups, while the OFC and temporal pole were activated only in patients (Table 3, Fig. 1). In controls, for the same contrast, a significant activation was also observed in the hippocampus and in visual areas (Table 3). For the same contrast in patients, an activation was also found in right associative temporal areas (BA 39), cerebellum and visual areas. However, no significant between-group difference for any of these regions was observed for that contrast.

**Table 3.** Functional magnetic resonance imaging activations during anticipation of loss *v*. neutral

		Healthy controls				Subjects with schizophrenia			
	BA	Peak *t* test value	Talairach coordinates			Peak *t* test value	Talairach coordinates		
			x	y	z		x	y	z
Right dorsal caudate		4.44^a^	12	12	17				
Right dorsal caudate		4.60^a^	−12	6	20				
Right dorsolateral prefrontal cortex	46	4.93	50	32	16	5.60	48	38	13
Right dorsolateral prefrontal cortex	46	4.74	50	43	7	5.04	42	15	20
Right dorsolateral prefrontal cortex	9					5.12	39	6	25
Right orbitofrontal cortex	47					4.87	39	22	−5
Left orbitofrontal cortex	47					4.95	−33	16	−5
Left orbitofrontal cortex	47					4.50	−24	22	−10
Right temporal pole	38					9.15	48	16	−18
Right middle temporal gyrus	21	6.54	59	−45	−2				
Right middle temporal gyrus	39					5.77	30	−55	26
Right occipital gyrus	19	6.24	24	−100	6	5.62	33	−76	24
Left precuneus/cuneus	19/18	7.00	−24	−97	1	5.59	−27	−75	30
Left superior occipital gyrus	19					4.77	−30	−81	24
Right cerebellar vermis						5.90	30	−63	−21
Right cerebellar vermis						4.77	30	−52	−22
Left cerebellar vermis						5.54	−18	−72	−18

BA, Brodmann area.

Brain activation during anticipation of loss. All results are significant at *p* < 0.05, false discovery rate corrected for multiple comparisons except when otherwise specified.

a Significant family-wise error corrected for small volume.

#### Feedback processing

Both patients and controls showed a significant activation of the ventral striatum during reward *v*. neutral feedback, which was significant only on the left side in patients and on both sides in controls (Table 4). Both groups activated the right OFC (BA 47), the key cortical region involved in reward valuation, and the adjacent right temporal pole (BA 38), functionally connected to the insula and OFC. Patients also activated the anterior insula (BA 13) bilaterally. Both groups activated the mPFC (BA 32) and the adjacent anterior cingulate (BA 24). Both groups activated right cerebellum and postcentral (BA 43) areas.

**Table 4.** Functional magnetic resonance imaging activations during feedback evaluation

		Reward *v*. neutral feedback							
		Healthy controls				Subjects with schizophrenia			
		Peak *t* test	Talairach coordinates			Peak *t* test	Talairach coordinates		
	BA	value	x	y	z	value	x	y	z
Right ventral striatum		4.09^a^	12	5	1				
Left ventral striatum		4.87^a^	−12	2	1	4.22^a^	−12	2	1
Left mesial prefrontal cortex	32	5.85	0	15	28	5.73	−3	9	34
Left anterior cingulate	24	6.81	−6	−5	34	5.10	0	18	36
Right orbitofrontal cortex	47	9.04	39	16	−3	4.64	39	14	−4
Right temporal pole	38	7.61	50	10	−10	4.46	56	13	−8
Right insula	13					5.63	39	−1	6
Left insula	13					8.65	−39	−1	1
Right cerebellar vermis		7.08	15	−55	−39	7.33	6	−61	−26
Left cerebellar vermis						5.96	0	−64	−36
Right postcentral gyrus	43	6.59	62	−17	21	6.53	62	−18	16

BA, Brodmann area.

Brain activation during reward feedback. All results are significant at *p* < 0.05, false discovery rate corrected for multiple comparisons except when otherwise specified.

a Significant family-wise error corrected for small volume.

No between-group difference was observed for any of these regions.

#### Correlations

Correlations between anhedonia and avolition were modest and not statistically significant (*r* = 0.38, *p* = 0.06) and the same was true for anhedonia and motivation (*r* = −0.29, *p* = 0.16). Furthermore, avolition and motivation scores were not correlated with TEPS score for anticipatory pleasure (*r* = −0.18, *p* = 0.387 and *r* = 0.19, *p* = 0.375, respectively). These results indicated that the constructs of anhedonia and avolition are at least partially independent and that avolition and motivation do not derive from a deficit in the ability to anticipate pleasure. On the other hand, avolition was significantly correlated to motivation in the real world (*r* = −0.66, *p* < 0.0001). This latter correlation indicates that avolition and motivation are intercorrelated constructs.

For the ventral striatum activity during anticipation, no significant correlation with anhedonia, motivation and avolition was found in patients. For the dorsal caudate activity during anticipation for the contrast reward *v*. neutral, a positive correlation (online Supplementary Fig. S3) was found with real-life motivation (*r* = 0.53, *p* < 0.006 for the left side; *r* = 0.61, *p* < 0.001 for the right side), and a negative correlation (online Supplementary Fig. S3) was observed with avolition (*r* = −0.50, *p* < 0.007 for the left side; *r* = −0.46, *p* < 0.01 for the right side; the latter did not survive Bonferroni correction). The correlation between the left dorsal caudate and avolition remained significant after controlling for the effects of PANSS depression and positive symptoms factors, as well as chlorpromazine equivalent dose (*r* = −0.53; *p* < 0.008).

Differences in the association of avolition/motivation with activity of the dorsal caudate or ventral striatum for the contrast reward *v*. neutral were tested using the Steiger test for dependent correlations and found to be statistically significant (*Z* = −1.98, *n* = 28, *p* < 0.05 for the correlations of avolition with left dorsal caudate and left ventral striatum; *Z* = 2.01, *n* = 27, *p* < 0.04 for the correlations of motivation with left dorsal caudate and left ventral striatum; *Z* = 2.81, *n* = 27, *p* < 0.005 for the correlations of motivation with right dorsal caudate and right ventral striatum.)

For the dorsal caudate activity during anticipation for the contrast loss *v*. neutral, no significant correlation was found with anhedonia, motivation and avolition.

No significant correlation was observed for the feedback analyses.

#### Comparisons of high- *v.* low-avolition subjects

A median split analysis was carried out to compare patients with high *v*. low avolition scores on ventral striatum and dorsal caudate activation and on demographic, psychometric and clinical variables.

High- and low-avolition subgroups did not differ on age (*F*_1,26_ = 0.71, *p* = 0.41), education (*F*_1,26_ = 0.46, *p* = 0.51) and IQ (*F*_1,26_ = 3.43, *p* = 0.08). The high-avolition group, in comparison with the low-avolition group, was receiving a higher chlorpromazine equivalent dose of antipsychotic (*F*_1,22_ = 4.94, *p* = 0.04). The chlorpromazine equivalent dose was entered as covariate when the two subgroups were compared on striatal activity. No difference was found between the two subgroups with respect to the scores on PANSS depression and positive symptom factors, TEPS and PAS. Real-life motivation was significantly lower in high- *v.* low-avolition patients (*F*_1,24_ = 9.55, *p* < 0.005).

ANCOVA on the ventral striatum activity (reward *v*. neutral condition) with group (DS, NDS, HC) as between factor and education and IQ as covariates did not show any significant main effect of group (*F*_2,43_ = 0.55, *p* = 0.58); the same analysis on the left dorsal caudate activity yielded a significant group effect (*F*_2,43_ = 2.83, *p* < 0.05) and Bonferroni *post-hoc* test revealed a significantly reduced activity only in the high-avolition subgroup *v*. HC (online Supplementary Fig. S4).

Comparison between the high- and low-avolition subgroups, with chlorpromazine equivalent dose as covariate, on the ventral striatum activity (reward *v*. neutral condition) did not show any significant main effect of group (*F*_1,25_ = 0.001, *p* < 0.97); while the same analysis on the left dorsal caudate activity yielded a significant group effect (*F*_1,25_ = 7.50, *p* < 0.03), due to lower activity in the high-avolition subgroup (online Supplementary Fig. S4).

#### Comparisons of DS *v.* NDS patients

Eleven patients were classified by the SDS as having DS (i.e. their negative symptoms were primary and persistent; avolition was one of the two or more negative symptoms justifying the diagnosis of DS in all 11 cases, and all of them were in the high-avolition group) and 17 as having NDS (only 3/17 were in the high-avolition group).

DS and NDS subgroups did not differ on age, education, PANSS depression and positive symptom factors, TEPS and chlorpromazine equivalent dose of antipsychotic. Compared with the NDS subgroup, DS patients had significantly lower IQ (*F*_1,26_ = 9.96, *p* < 0.004), higher PAS (*F*_1,23_ = 15.08, *p* < 0.0008) scores and, as expected, higher avolition (*F*_1,23_ = 46.12, *p* < 0.000001) and lower motivation (*F*_1,23_ = 23.65, *p* < 0.00007) scores. IQ and PAS scores were entered as covariate when comparing the two subgroups on striatal activity.

ANCOVA on the ventral striatum activity (reward *v*. neutral condition) with group (DS, NDS and HC) as between factor and education and IQ as covariates did not show any significant main effect of group (*F*_2,43_ = 0.63, *p* = 0.54). ANCOVA on the left dorsal caudate activity for the same contrast yielded a significant group effect (*F*_2,43_ = 2.65, *p* < 0.05); Bonferroni *post-hoc* test revealed a significantly reduced activity only in the DS subgroup *v*. HC (online Supplementary Fig. S5).

Comparison between the two subgroups of patients on the ventral striatum activity for the same contrast, with PAS scores and IQ as covariates, did not show a significant group effect (*F*_1,24_ = 3.14, *p* = 0.10) (online Supplementary Fig. S5); while the same analysis on the left dorsal caudate activity (for the same contrast and with the same covariates) yielded a significant group effect (*F*_1,24_ = 3.59, *p* < 0.05), due to lower activity in the DS subgroup (online Supplementary Fig. S5).

## Discussion

The present study aimed to assess the role of ventral striatum and dorsal caudate in motivation deficits of individuals with schizophrenia. To this aim, we investigated: (*a*) the activation of these two regions during processing of reward or loss in persons with schizophrenia living in the community and stabilized on treatment with SGAs only, and (*b*) the relationships of ventral striatum and dorsal caudate activation with hedonic experience and motivation.

Patients with schizophrenia did not differ from HC with respect to their hedonic experience. This conclusion is based on the PAS and TEPS comparisons, but also on findings concerning ventral striatum activation during reward feedback, that was observed in both patients and controls, and was comparable between them. This is in line with previous reports of intact in-the-moment ability to experience pleasure, in the presence of impaired capacity to translate pleasurable experiences into motivational states, in schizophrenia patients (see Strauss *et al.* 2013*a* for a review). Our findings of preserved ventral striatal activation in patients with schizophrenia stabilized on SGAs are in line with several previous studies (Schlagenhauf *et al.* 2008; Walter *et al.* 2009; Simon *et al.* 2010; Waltz *et al.* 2010; Nielsen *et al.* 2012*a*, *b*). Some authors (Waltz *et al.* 2010) hypothesized that the personalized performance in the MID task produced negative prediction errors (NPEs, i.e. more reward than expected) more frequently than positive prediction errors (PPEs, i.e. less reward than expected), and response of the striatum to NPEs might be largely intact in patients; however, in drug-naive subjects or in those treated with FGAs, a deficit of ventral striatal activity was found using the same task. Other authors speculated that SGAs might normalize at least in part ventral striatal response, as demonstrated by longitudinal studies, while FGAs do not (Juckel *et al.* 2006*b*; Schlagenhauf *et al.* 2008; Walter *et al.* 2009; Nielsen *et al.* 2012*a*, *b*). It has been hypothesized that sparing of reward processing and ventral striatum response observed with SGAs is related to their fast dissociation from D_2_ receptors and low potential to induce extrapyramidal side-effects and depression, which might cause secondary negative symptoms (Juckel *et al.* 2006*a*,*b*, Abler *et al.* 2008; Schlagenhauf *et al.* 2008; Walter *et al.* 2009; Waltz *et al.* 2010; Nielsen *et al.* 2012*a*,*b*).

The hypothesis that a deficit of reward anticipation due to hypoactivation of the ventral striatum is responsible for the motivation deficit is not supported by our findings. We found an activation of ventral striatum and key cortical regions involved in reward anticipation in both patients and controls (including the OFC, regarded as a key region in processing receipt of monetary reward; Knutson *et al.* 2003) and no association between the activation of these regions during reward anticipation and measures of real-life motivation or avolition. The discrepancy with previous studies reporting an association between ventral striatal activation and avolition (also in the absence of a significant reduction of ventral striatal activation in patients compared to controls) (e.g. Simon *et al.* 2010; Waltz *et al.* 2010) might be explained by the use of different measures of avolition, e.g. the sum of anhedonia and avolition scores (Waltz *et al.* 2010) or differences in the severity of anhedonia, which might contribute to motivation deficits through a reduced sensitivity to reward (Simon *et al.* 2010). As a matter of fact, in the study of Simon *et al.* (2010), patients differed from HC in the severity of physical anhedonia, while our patients did not differ on the same measure from controls. The absence of significant degrees of anhedonia (as assessed with both the PAS and TEPS) in our sample might also explain the lack of significant associations between anhedonia and ventral striatal activation in our results.

The OFC, the key cortical region involved in reward processing, in particular in reward value coding (Peters & Büchel, 2010; Sescousse *et al.* 2010), was active in both patients and controls, and this activation did not show a significant correlation with either hedonic experience or motivation measures.

A deficit of motivation may also reflect hypofunctioning of the brain network including the dorsal caudate and DLPFC. In fact, an activation of the dorsal caudate was found in studies in which subjects’ action was essential for the outcome, such as those using the MID task, in which the outcome depends on subjects’ speeded response (Delgado *et al.* 2000, 2004; Elliott *et al.* 2000; Knutson *et al*. 2000, 2001*a*, *b*; Tricomi *et al.* 2004; Grahn *et al.* 2008), demonstrating that the dorsal caudate is sensitive to action-outcome contingency, rather than to rewards in themselves (Grahn *et al.* 2008). In addition, evidence has been provided by neuroimaging and neuropsychological investigations showing that the dorsal caudate is implicated in different aspects of motivational processes with respect to ventral striatum (Yin *et al.* 2006; Yin & Knowlton, 2006; Balleine *et al.* 2007). Recently, in healthy subjects, the dorsal striatum was shown to be active when subjects showed increased motivation, independent of reward anticipation, while ventral striatum activation was observed during reward anticipation only (Miller *et al.* 2014).

The role of the DLPFC in decision-making based on reward values and effort calculations, i.e. other aspects of motivation, is also supported by several experimental findings (Miller & Cohen, 2001; Manes *et al.* 2002).

In our study, the dorsal caudate was significantly activated in controls for the anticipation of both reward and loss, while in persons with schizophrenia it showed no activation for either condition. The activity of the dorsal caudate during reward anticipation was significantly associated with real-life motivation and avolition, but not with anhedonia measures. A median split analysis confirmed the link between avolition and dorsal caudate hypoactivation in patients, as the dorsal caudate activity was significantly reduced in high-avolition as compared to both HC and low-avolition patients. No other assessed variable could explain the finding, as the two subgroups of patients did not differ for any demographic, psychometric or clinical variables, except chlorpromazine equivalents, that were used as covariate in all comparisons between the two groups of patients. Our analyses concerning DS and NDS subgroups further corroborate the association of dorsal caudate activity with avolition: all DS subjects were in the high-avolition group while only 3/17 of the NDS group were in the same group. DS patients, characterized by a greater severity of avolition and reduced motivation, had a reduced dorsal caudate activity for the contrast reward *v*. neutral *v*. both HC and NDS subjects. Furthermore, DS patients had comparable activation of the ventral striatum for the contrast reward *v*. neutral with respect to both HC and NDS subjects. The higher social anhedonia and lower IQ of DS *v*. NDS were used as covariates and could not account for the reduced dorsal caudate activity.

In line with a report showing an inverse association of the activity of the dorsal striatum, but not of the DLPFC, with negative symptom severity in schizophrenia patients (Ehrlich *et al.* 2012), we found no deficit in the activation of DLPFC and no association between the degree of its activation and the severity of motivation deficits in our patients. As the activity of this region underlies specific aspects of reward processing, i.e. the ability to generate and execute goal-directed action plans necessary to achieve a valued outcome, our findings suggest that this aspect of reward processing may not be the one primarily affected in motivational deficits of patients with schizophrenia stabilized on treatment with SGAs. This tentative conclusion is in line with findings from a meta-analysis of fMRI studies in schizophrenia, in which Goghari *et al.* (2010) reported that negative symptoms have no consistent relationship with DLPFC activity, as well as with previous structural MRI findings from our group (Volpe *et al.* 2012) showing no association between DLPFC gray-matter volume and negative symptoms in schizophrenia subjects.

An activation of the right hippocampus and parahippocampal gyrus was also observed in HC. Actually, the input from these structures to the striatum is important to integrate information related to reward processing and memory (O'Donnell & Grace, 1995). Patients demonstrated no activation in these regions, but activated a larger number of cortical areas, which might reflect an attempt to overcome the hypoactivity of the dorsal caudate and connected regions, such as the hippocampus, and achieve normal performance through the involvement of alternative circuits.

The main strengths of the present study include the selection of patients with schizophrenia treated with SGAs only, the use of specific instruments for the independent assessment of motivation and hedonic experience, the assessment of different aspects of reward processing, and the focus on both the ventral striatum and the dorsal caudate.

As to limitations, the possibility that low IQ in some of our patients represented a confounding factor cannot be entirely ruled out. However, it is unlikely that learning deficits or other cognitive impairments contributed to our findings. Indeed, IQ was used as a covariate in data analyses; the task was overly simple and did not imply learning (subjects practiced the task in advance); and task difficulty was personalized for each participant, so that they could succeed on at least 66% of the trials.

Treatment of patients with antipsychotic drugs might represent a further limitation, as in the patient group the lack of dorsal striatum activation during reward anticipation might be attributed to the treatment with SGAs. However, antipsychotics would be expected to dampen the response to reward in all sections of the striatum, since all of them receive dopamine innervation, while we observed in our patients a significant activation of the ventral striatum during reward anticipation, a pattern similar to that observed in healthy subjects. These results argue against a role of SGAs in our findings.

Another limitation is the assessment of extrapyramidal symptoms only during the routine neurological examination of our patients, without using a standardized instrument. Extrapyramidal symptoms might cause secondary negative symptoms which might confound results concerning avolition. However, we think it is unlikely that extrapyramidal symptoms affected our findings concerning avolition. First, subjects with primary and persistent negative symptoms (deficit schizophrenia), identified using the SDS, were the majority of the subjects with high avolition and, by definition, the influence of extrapyramidal symptoms on avolition in this group had to be excluded. Second, our subjects had a preserved ventral striatal response to reward, that would be unlikely in the presence of extrapyramidal side-effects.

Finally, to measure real-life motivation, we used an index derived from the QLS scale, and healthy subjects showed a ceiling effect on this instrument, preventing the exploration of correlations between motivation and striatal activation in this group.

In conclusion, our findings support the notion that hedonic and motivational aspects of reward are subtended by different subdivisions of the striatum; that avolition in schizophrenia emerges independently of in-the-moment ability to experience pleasure, and that it is not linked to a ventral striatum dysfunction (at least in patients treated with SGAs) but to the hypoactivation of the dorsal caudate.

Our finding of a dorsal striatum hypoactivation in patients with schizophrenia is of interest also in the light of the recently documented role of the human dorsal striatum in complex social tasks, such as social interactions in situations requiring cooperation (Rilling *et al.* 2002) or revenge (de Quervain *et al.* 2004), or acquisition of social reputations through trial and error (King-Casas *et al.* 2005). A future challenge for research in schizophrenia might be to further improve our understanding of the role of the dorsal striatum in motivation, using various reward and social interaction paradigms. Progress in this field might foster the development of innovative pharmacological and rehabilitation treatments for schizophrenia.

## Supplementary material

For supplementary material accompanying this paper visit http://dx.doi.org/10.1017/S0033291714002943.

## References

[ref1] Abler B, Greenhouse I, Ongur D, Walter H, Heckers S (2008). Abnormal reward system activation in mania. Neuropsychopharmacology 33, 2217–2227. 17987058 10.1038/sj.npp.1301620 PMC2574988

[ref2] Balleine BW, Delgado MR, Hikosaka O (2007). The role of the dorsal striatum in reward and decision-making. Journal of Neuroscience 27, 8161–8165. 17670959 10.1523/JNEUROSCI.1554-07.2007 PMC6673072

[ref3] Balleine BW, O'Doherty JP (2010). Human and rodent homologies in action control: corticostriatal determinants of goal-directed and habitual action. Neuropsychopharmacology 35, 48–69. 19776734 10.1038/npp.2009.131 PMC3055420

[ref4] Barch DM, Dowd EC (2010). Goal representations and motivational drive in schizophrenia: the role of prefrontal-striatal interactions. Schizophrenia Bulletin 36, 919–934. 20566491 10.1093/schbul/sbq068 PMC2930335

[ref5] Berridge KC, Robinson TE (2003). Parsing reward. Trends in Neurosciences 26, 507–513. 12948663 10.1016/S0166-2236(03)00233-9

[ref6] Cohen AS, Minor KS (2010). Emotional experience in patients with schizophrenia revisited: meta-analysis of laboratory studies. Schizophrenia Bulletin 36, 143–150. 18562345 10.1093/schbul/sbn061 PMC2800132

[ref7] Chapman LJ, Chapman JP (1978). Revised Physical Anhedonia Scale. Available from L. J. Chapman, Department of Psychology, 1202 West Johnson Street, University of Wisconsin, Madison, WI 53706.

[ref8] Delgado MR, Miller MM, Inati S, Phelps EA (2005). An fMRI study of reward-related probability learning. Neuroimage 24, 862–873. 15652321 10.1016/j.neuroimage.2004.10.002

[ref9] Delgado MR, Nystrom LE, Fissell C, Noll DC, Fiez JA (2000). Tracking the hemodynamic responses to reward and punishment in the striatum. Journal of Neurophysiology 84, 3072–3077. 11110834 10.1152/jn.2000.84.6.3072

[ref10] Delgado MR, Stenger VA, Fiez JA (2004). Motivation-dependent responses in the human caudate nucleus. Cerebral Cortex 14, 1022–1030. 15115748 10.1093/cercor/bhh062

[ref11] de Quervain DJ, Fischbacher U, Treyer V, Schellhammer M, Schnyder U, Buck A, Fehr E (2004). The neural basis of altruistic punishment. Science 27, 1254–1258. 15333831 10.1126/science.1100735

[ref12] Der-Avakian A, Markou A (2012). The neurobiology of anhedonia and other reward-related deficits. Trends in Neurosciences 35, 68–77. 22177980 10.1016/j.tins.2011.11.005 PMC3253139

[ref13] Dolan RJ, Dayan P (2013). Goals and habits in the brain. Neuron 80, 312–325. 24139036 10.1016/j.neuron.2013.09.007 PMC3807793

[ref14] Ehrlich S, Yendiki A, Greve DN, Manoach DS, Ho BC, White T, Schulz SC, Goff DC, Gollub RL, Holt DJ (2012). Striatal function in relation to negative symptoms in schizophrenia. Psychological Medicine 42, 267–282. 21733291 10.1017/S003329171100119X

[ref15] Elliott R, Friston KJ, Dolan RJ (2000). Dissociable neural responses in human reward systems. Journal of Neuroscience 20, 6159–6165. 10934265 10.1523/JNEUROSCI.20-16-06159.2000 PMC6772605

[ref16] Esslinger C, Englisch S, Inta D, Rausch F, Schirmbeck F, Mier D, Kirsch P, Meyer-Lindenberg A, Zink M (2012). Ventral striatal activation during attribution of stimulus saliency and reward anticipation is correlated in unmedicated first episode schizophrenia patients. Schizophrenia Research 140, 114–121. 22784688 10.1016/j.schres.2012.06.025

[ref17] Foussias G, Mann S, Zakzanis KK, van Reekum R, Remington G (2009). Motivational deficits as the central link to functioning in schizophrenia: a pilot study. Schizophrenia Research 115, 333–337. 19836211 10.1016/j.schres.2009.09.020

[ref18] Foussias G, Remington G (2010). Negative symptoms in schizophrenia: avolition and Occam's razor. Schizophrenia Bulletin 36, 359–369. 18644851 10.1093/schbul/sbn094 PMC2833114

[ref19] Galderisi S, Bucci P, Mucci A, Kirkpatrick B, Pini S, Rossi A, Vita A, Maj M (2013). Categorical and dimensional approaches to negative symptoms of schizophrenia: focus on long-term stability and functional outcome. Schizophrenia Research 147, 157–162. 23608244 10.1016/j.schres.2013.03.020

[ref20] Gard DE, Gard MG, Kring AM, John OP (2006). Anticipatory and consummatory components of the experience of pleasure: a scale development study. Journal of Research in Personality 40, 1086–1102.

[ref21] Gard DE, Kring AM, Gard MG, Horan WP, Green MF (2007). Anhedonia in schizophrenia: distinctions between anticipatory and consummatory pleasure. Schizophrenia Research 93, 253–260. 17490858 10.1016/j.schres.2007.03.008 PMC1986826

[ref22] Gardner DM, Murphy AL, O'Donnell H, Centorrino F, Baldessarini RJ (2010). International consensus study of antipsychotic dosing. American Journal of Psychiatry 167, 686–693. 20360319 10.1176/appi.ajp.2009.09060802

[ref23] Goghari VM, Sponheim SR, MacDonald AW 3rd (2010). The functional neuroanatomy of symptom dimensions in schizophrenia: a qualitative and quantitative review of a persistent question. Neuroscience Biobehavioral Review 34, 468–86. 10.1016/j.neubiorev.2009.09.004 PMC2813961 19772872

[ref24] Grahn JA, Parkinson JA, Owen AM (2008). The cognitive functions of the caudate nucleus. Progress in Neurobiology 86, 141–155. 18824075 10.1016/j.pneurobio.2008.09.004

[ref25] Haber SN, Knutson B (2010). The reward circuit: linking primate anatomy and human imaging. Neuropsychopharmacology 35, 4–26. 19812543 10.1038/npp.2009.129 PMC3055449

[ref26] Haruno M, Kawato M (2006). Different neural correlates of reward expectation and reward expectation error in the putamen and caudate nucleus during stimulus-action-reward association learning. Journal of Neurophysiology 95, 948–959. 16192338 10.1152/jn.00382.2005

[ref27] Heerey EA, Gold JM (2007). Patients with schizophrenia demonstrate dissociation between affective experience and motivated behavior. Journal of Abnormal Psychology 116, 268–278. 17516760 10.1037/0021-843X.116.2.268

[ref28] Heerey EA, Robinson BM, McMahon RP, Gold JM (2007). Delay discounting in schizophrenia. Cognitive Neuropsychiatry 12, 213–221. 17453902 10.1080/13546800601005900 PMC3746343

[ref29] Heinrichs DW, Hanlon TE, Carpenter WT Jr. (1984). The Quality of Life Scale: an instrument for rating the schizophrenic deficit syndrome. Schizophrenia Bulletin 10, 388–398. 6474101 10.1093/schbul/10.3.388

[ref30] Juckel G, Schlagenhauf F, Koslowski M, Filonov D, Wüstenberg T, Villringer A, Knutson B, Kienast T, Gallinat J, Wrase J, Heinz A (2006 *b*). Dysfunction of ventral striatal reward prediction in schizophrenic patients treated with typical, not atypical, neuroleptics. Psychopharmacology 187, 222–228. 16721614 10.1007/s00213-006-0405-4

[ref31] Juckel G, Schlagenhauf F, Koslowski M, Wüstenberg T, Villringer A, Knutson B, Wrase J, Heinz A (2006 *a*). Dysfunction of ventral striatal reward prediction in schizophrenia. Neuroimage 29, 409–416. 16139525 10.1016/j.neuroimage.2005.07.051

[ref32] Kay SR, Fiszbein A, Opler LA (1987). The Positive and Negative Syndrome Scale (PANSS) for schizophrenia. Schizophrenia Bulletin 13, 261–276. 3616518 10.1093/schbul/13.2.261

[ref33] Kimhy D, Yale S, Goetz RR, McFarr LM, Malaspina D (2006). The factorial structure of the Schedule for the Deficit Syndrome in Schizophrenia. Schizophrenia Bulletin 32, 274–278. 16177274 10.1093/schbul/sbi064 PMC2632208

[ref34] King-Casas B, Tomlin D, Anen C, Camerer CF, Quartz SR, Montague PR (2005). Getting to know you: reputation and trust in a two-person economic exchange. Science 308, 78–83. 15802598 10.1126/science.1108062

[ref35] Kirkpatrick B, Buchanan RW, McKenney PD, Alphs LD, Carpenter WT Jr. (1989). The Schedule for the Deficit Syndrome: an instrument for research in schizophrenia. Psychiatry Research 30, 119–123. 2616682 10.1016/0165-1781(89)90153-4

[ref36] Kirkpatrick B, Fenton WS, Carpenter WT Jr., Marder SR (2006). The NIMH-MATRICS consensus statement on negative symptoms. Schizophrenia Bulletin 32, 214–219. 16481659 10.1093/schbul/sbj053 PMC2632223

[ref37] Knutson B, Adams CM, Fong GW, Hommer D (2001 *b*). Anticipation of increasing monetary reward selectively recruits nucleus accumbens. Journal of Neuroscience 21, RC159. 10.1523/JNEUROSCI.21-16-j0002.2001 PMC6763187 11459880

[ref38] Knutson B, Fong GW, Adams CM, Varner JL, Hommer D (2001 *a*). Dissociation of reward anticipation and outcome with event-related fMRI. Neuroreport 12, 3683–3687. 11726774 10.1097/00001756-200112040-00016

[ref39] Knutson B, Fong GW, Bennett SM, Adams CM, Hommer D (2003). A region of mesial prefrontal cortex tracks monetarily rewarding outcomes: characterization with rapid event-related fMRI. Neuroimage 18, 263–272. 12595181 10.1016/s1053-8119(02)00057-5

[ref40] Knutson B, Westdorp A, Kaiser E, Hommer D (2000). FMRI visualization of brain activity during a monetary incentive delay task. Neuroimage 12, 20–27. 10875899 10.1006/nimg.2000.0593

[ref41] Kring AM, Moran EK (2008). Emotional response deficits in schizophrenia: insights from affective science. Schizophrenia Bulletin 34, 819–834. 18579556 10.1093/schbul/sbn071 PMC2632476

[ref42] Manes F, Sahakian B, Clark L, Antoun N, Aitken M, Robbins T (2002). Decision-making processes following damage to the prefrontal cortex. Brain 125, 624–639. 11872618 10.1093/brain/awf049

[ref43] Mann CL, Footer O, Chung YS, Driscoll LL, Barch DM (2013). Spared and impaired aspects of motivated cognitive control in schizophrenia. Journal of Abnormal Psychology 122, 745–755. 23834064 10.1037/a0033069 PMC3863584

[ref44] Miller EK, Cohen JD (2001). An integrative theory of prefrontal cortex function. Annual Review of Neuroscience 21, 167–202. 10.1146/annurev.neuro.24.1.167 11283309

[ref45] Miller EM, Shankar MU, Knutson B, McClure SM (2014). Dissociating motivation from reward in human striatal activity. Journal of Cognitive Neuroscience 26, 1075–1084. 24345173 10.1162/jocn_a_00535 PMC5808846

[ref46] Nakagami E, Hoe M, Brekke JS (2010). The prospective relationships among intrinsic motivation, neurocognition, and psychosocial functioning in schizophrenia. Schizophrenia Bulletin 36, 935–948. 20462998 10.1093/schbul/sbq043 PMC2930331

[ref47] Nielsen MØ, Rostrup E, Wulff S, Bak N, Broberg BV, Lublin H, Kapur S, Glenthøj B (2012 *b*). Improvement of brain reward abnormalities by antipsychotic monotherapy in schizophrenia. Archives of General Psychiatry 69, 1195–1204. 22868877 10.1001/archgenpsychiatry.2012.847

[ref48] Nielsen MØ, Rostrup E, Wulff S, Bak N, Lublin H, Kapur S, Glenthøj B (2012 *a*). Alterations of the brain reward system in antipsychotic naïve schizophrenia patients. Biological Psychiatry 71, 898–905. 22418013 10.1016/j.biopsych.2012.02.007

[ref49] O'Donnell P, Grace AA (1995). Synaptic interactions among excitatory afferents to nucleus accumbens neurons: hippocampal gating of prefrontal cortical input. Journal of Neuroscience 15, 3622–3639. 7751934 10.1523/JNEUROSCI.15-05-03622.1995 PMC6578219

[ref50] Palmiter RD (2008). Dopamine signaling in the dorsal striatum is essential for motivated behaviors: lessons from dopamine-deficient mice. Annals of the New York Academy of Sciences 1129, 35–46. 18591467 10.1196/annals.1417.003 PMC2720267

[ref51] Peters J, Büchel C (2010). Neural representations of subjective reward value. Behavioural Brain Research 213, 135–141. 20420859 10.1016/j.bbr.2010.04.031

[ref52] Rilling J, Gutman D, Zeh T, Pagnoni G, Berns G, Kilts C (2002). A neural basis for social cooperation. Neuron 18, 395–405. 12160756 10.1016/s0896-6273(02)00755-9

[ref53] Robinson JL, Laird AR, Glahn DC, Blangero J, Sanghera MK, Pessoa L, Fox PM, Uecker A, Friehs G, Young KA, Griffin JL, Lovallo WR, Fox PT (2012). The functional connectivity of the human caudate: an application of meta-analytic connectivity modeling with behavioral filtering. Neuroimage 60, 117–129. 22197743 10.1016/j.neuroimage.2011.12.010 PMC3288226

[ref54] Schlagenhauf F, Juckel G, Koslowski M, Kahnt T, Knutson B, Dembler T, Kienast T, Gallinat J, Wrase J, Heinz A (2008). Reward system activation in schizophrenic patients switched from typical neuroleptics to olanzapine. Psychopharmacology 196, 673–684. 18097655 10.1007/s00213-007-1016-4

[ref55] Schlagenhauf F, Sterzer P, Schmack K, Ballmaier M, Rapp M, Wrase J, Juckel G, Gallinat J, Heinz A (2009). Reward feedback alterations in unmedicated schizophrenia patients: relevance for delusions. Biological Psychiatry 65, 1032–1039. 19195646 10.1016/j.biopsych.2008.12.016

[ref56] Sescousse G, Redouté J, Dreher JC (2010). The architecture of reward value coding in the human orbitofrontal cortex. Journal of Neuroscience 30, 13095–13104. 20881127 10.1523/JNEUROSCI.3501-10.2010 PMC6633499

[ref57] Sheehan DV, Lecrubier Y, Sheehan KH, Amorim P, Janavs J, Weiller E, Hergueta T, Baker R, Dunbar GC (1998). The Mini-International Neuropsychiatric Interview (M.I.N.I.): the development and validation of a structured diagnostic psychiatric interview for DSM-IV and ICD-10. Journal of Clinical Psychiatry 59(Suppl. 20), 22–33. 9881538

[ref58] Simon JJ, Biller A, Walther S, Roesch-Ely D, Stippich C, Weisbrod M, Kaiser S (2010). Neural correlates of reward processing in schizophrenia: relationship to apathy and depression. Schizophrenia Research 118, 154–161. 20005675 10.1016/j.schres.2009.11.007

[ref59] Simpson EH, Waltz JA, Kellendonk C, Balsam PD (2012). Schizophrenia in translation: dissecting motivation in schizophrenia and rodents. Schizophrenia Bulletin 38, 1111–1117. 23015686 10.1093/schbul/sbs114 PMC3494038

[ref60] Strauss GP, Horan WP, Kirkpatrick B, Fischer BA, Keller WR, Miski P, Buchanan RW, Green MF, Carpenter WT Jr. (2013 *b*). Deconstructing negative symptoms of schizophrenia: avolition-apathy and diminished expression clusters predict clinical presentation and functional outcome. Journal of Psychiatric Research 47, 783–790. 23453820 10.1016/j.jpsychires.2013.01.015 PMC3686506

[ref61] Strauss GP, Waltz JA, Gold JM (2013 *a*). A review of reward processing and motivational impairment in schizophrenia. Schizophrenia Bulletin. Published online: 22 2 2014. doi:10.1093/schbul/sbt197. PMC3934394 24375459

[ref62] Tricomi EM, Delgado MR, Fiez JA (2004). Modulation of caudate activity by action contingency. Neuron 41, 281–292. 14741108 10.1016/s0896-6273(03)00848-1

[ref63] Volpe U, Mucci A, Quarantelli M, Galderisi S, Maj M (2012). Dorsolateral prefrontal cortex volume in patients with deficit or nondeficit schizophrenia. Progress in Neuro-psychopharmacology and Biological Psychiatry 37, 264–269. 22349577 10.1016/j.pnpbp.2012.02.003

[ref64] Walter H, Kammerer H, Frasch K, Spitzer M, Abler B (2009). Altered reward functions in patients on atypical antipsychotic medication in line with the revised dopamine hypothesis of schizophrenia. Psychopharmacology 206, 121–132. 19521678 10.1007/s00213-009-1586-4

[ref65] Waltz JA, Frank MJ, Robinson BM, Gold JM (2007). Selective reinforcement learning deficits in schizophrenia support predictions from computational models of striatal-cortical dysfunction. Biological Psychiatry 62, 756–764. 17300757 10.1016/j.biopsych.2006.09.042 PMC2083701

[ref66] Waltz JA, Schweitzer JB, Ross TJ, Kurup PK, Salmeron BJ, Rose EJ, Gold JM, Stein EA (2010). Abnormal responses to monetary outcomes in cortex, but not in the basal ganglia, in schizophrenia. Neuropsychopharmacology 35, 2427–2439. 20720534 10.1038/npp.2010.126 PMC2955756

[ref67] Wallis JD (2007). Orbitofrontal cortex and its contribution to decision-making. Annual Review of Neuroscience 30, 31–56. 10.1146/annurev.neuro.30.051606.094334 17417936

[ref68] Wang AY, Miura K, Uchida N (2013). The dorsomedial striatum encodes net expected return, critical for energizing performance vigor. Nature Reviews Neuroscience 16, 639–647. 10.1038/nn.3377 PMC3651899 23584742

[ref69] Yin HH, Knowlton BJ (2006). The role of the basal ganglia in habit formation. Nature Reviews Neuroscience 7, 464–476. 10.1038/nrn1919 16715055

[ref70] Yin HH, Knowlton BJ, Balleine BW (2006). Inactivation of dorsolateral striatum enhances sensitivity to changes in the action-outcome contingency in instrumental conditioning. Behavioural Brain Research 166, 189–196. 16153716 10.1016/j.bbr.2005.07.012

